# A Qualitative Comparison of Different Logical Topologies for Wireless Sensor Networks

**DOI:** 10.3390/s121114887

**Published:** 2012-11-05

**Authors:** Quazi Mamun

**Affiliations:** School of Computing and Mathematics, Charles Sturt University, New South Wales 2678, Australia; E-Mail: qmamun@csu.edu.au; Tel./Fax: +61-269-334-725

**Keywords:** logical topology, performance metric, flat topology, cluster topology, tree topology, chain topology

## Abstract

Wireless Sensor Networks (WSNs) are formed by a large collection of power-conscious wireless-capable sensors without the support of pre-existing infrastructure, possibly by unplanned deployment. With a sheer number of sensor nodes, their unattended deployment and hostile environment very often preclude reliance on physical configuration or physical topology. It is, therefore, often necessary to depend on the logical topology. Logical topologies govern how a sensor node communicates with other nodes in the network. In this way, logical topologies play a vital role for resource-constraint sensor networks. It is thus more intuitive to approach the constraint minimizing problems from (logical) topological point of view. Hence, this paper aims to study the logical topologies of WSNs. In doing so, a set of performance metrics is identified first. We identify various logical topologies from different application protocols of WSNs, and then compare the topologies using the set of performance metrics.

## 1. Introduction

In Wireless Sensor Networks (WSNs), topology plays a vital role in minimizing various constraints, such as limited energy, latency, computational resource crisis, and quality of communication. For example, energy consumption is proportional to the number of packets sent or received. The receiving cost depends on packet size, while the transmission energy depends on the distance between the nodes. As topology inherently defines the type of routing paths, indicates whether to use broadcast or unicast, determines the sizes and types of packets and other overheads, choosing the right topology helps to reduce the amount of communication needed for a particular problem and thus save energy. An efficient topology, which ensures that neighbours are at a minimal distance, reduces the probability of message being lost between sensors. A topology can also reduce the radio interference, thus reducing the waiting time for sensors to transmit data. Moreover, topology facilitates data aggregation, which greatly reduces the amount of processing cycles and energy, thus giving a longer lifetime for the network.

In addition, topology inherently defines the size of a group, how to manage new members in a group, or how to deal with members who have left the group. With the awareness of the underlying network topology, more efficient routing or broadcasting schemes can be achieved. Furthermore, the network topology in WSNs can be changed by varying the nodes' transmitting ranges and also by adjusting the wake/sleep schedule of the nodes. Therefore, more energy can be saved if the network topology is maintained in an optimal manner.

All the discussions and examples above infer that logical topology plays a vital role for wireless networks, especially for resource-constraint WSNs. Moreover, logical topology facilitates WSNs in many ways to overcome different constraints such as minimizing energy consumption, maximizing lifetime, reducing interference, making networks scalable, *etc*. Thus, it is very crucial to compare different topologies and to choose the best topology while designing protocols or algorithms for WSNs. To do so, in this paper, we compare various topologies of wireless sensor networks using various performance metrics.

This paper primarily consists of two parts. In the first part, we provide detailed descriptions of existing topologies, which are identified from different protocols for WSNs. To design various protocols for WSNs, different underlying logical topologies have been used. Each topology has its own advantages and disadvantages under a specific working environment. Because of this, to compare and evaluate the effectiveness of each topology, a set the performance evaluation metrics is required. In doing so, this paper also focuses on two major issues, namely (i) the system model of the WSN, which would be used throughout the paper, and (ii) the list of performance metrics to evaluate existing topologies.

The second part of this paper presents a comparative discussion of performance of different existing topologies of WSNs. In doing so, various performance metrics are first identified, and then detailed comparisons among identified topologies are provided for each of these metrics. The paper ends with a table, which shows the comparison summary of the performance evaluation of different topologies.

## 2. System Model and Assumptions

As WSNs are very much application dependent, first of all we like to assert the types of WSNs applications for which the research applies. There are different types of application classifications for WSNs. One of the possible classifications of WSNs applications distinguishes applications according to the type of data that must be gathered in the network. Almost any application, in fact, could be classified into two categories: event detection (ED) and spatial process estimation (SPE) [1]. In ED, sensors are deployed to detect an event, for example a fire in a forest, a quake, *etc.* [2–4], whereas in SPE the WSNs aim at estimating a given physical phenomenon (e.g., the atmospheric pressure in a wide area, or the ground temperature variations in a small volcanic site), which can be modelled as a bi-dimensional random process. In this case, the main issue is to obtain the estimation of the entire behaviour of the spatial process based on the samples taken by sensors that are typically placed in random positions [5–8].

Now, let us describe the basic system model. This is a very familiar system model, and almost any application of event detection (ED) category or spatial process estimation (SPE) category can use this model. Moreover, the same model has been used by many researchers in designing various protocols for WSNs [9–13]. This system model has been used throughout this paper.

**Figure 1.**
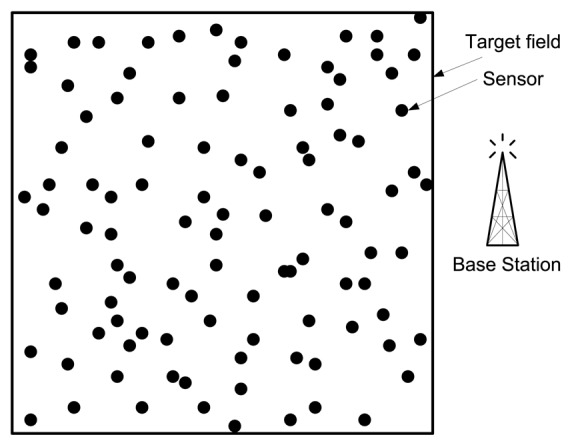
A system model of WSNs.

Wireless sensor networks are very much application and system model dependent. Algorithms/protocols that are designed based on one system model usually do not produce the same results or show the same effectiveness when they are applied on another system model without modification. Thus, it is important to define the system model before presenting any algorithm/protocol/architecture. The assumptions of the basic system model, which would be used throughout the paper, are described below:

- Assume a large-scale WSN. Large-scale WSNs consist of hundreds to thousands of nodes [14,15].
- They link the physical world to global communication networks for a broad set of applications. Assume a target field, where a large number of wireless sensor nodes are deployed randomly. The deployed sensors sense the data and send it to the base station periodically. The base station is located outside the target field (see Figure 1).
- Both the base station and the sensors are stationary after deployment.
- All sensor nodes have limited battery power, and recharge of the batteries is impossible. Efficient energy-aware protocols are thus required for energy conservation.
- All the sensors are homogeneous. They have the same initial power and communication and processing capabilities.
- All the sensors have limited sensing range. However, the sensors have the ability to control the transmission range depending on the distance between a sensor node and its next-hop node.
- The radio channel is symmetric such that energy required for transmitting a message from a sensor node A to another sensor node B is the same as the energy required for transmitting a message from the sensor node B to the sensor node A.
- All sensor nodes are sensing the environment at a fixed rate, and thus always have data to send to the end user.

## 3. A Review of Evaluation Criteria

In order to establish a standard set of evaluation criteria, it is important to describe different metrics, which would be used to evaluate the performance of different topologies. Interestingly, it is found that the set of evaluation criteria and their definitions vary quite considerably. The following six sub-sections describe these metrics.

### 3.1. Evaluation Measures Related to Energy Usage

Not surprisingly, given the intrinsic constraints of WSNs, almost all evaluation strategies include some form of energy metric. Different energy related metrics that are used by the researchers are listed below.

- The metric most commonly used to evaluate the efficiency of the sensor network is the overall energy consumption. This is measured by adding the energy dissipation by each sensor in the network.
- Another important metric is energy distribution. This is a qualitative metric, rather than quantitative one. This metric measures how evenly the energy dissipation is distributed. This is important for the sensor network to balance the energy consumption by the participating sensor nodes. If some sensors dissipate energy rapidly compared with other group of sensors, *i.e.*, energy consumption is not evenly distributed, this adversely affects the system lifetime of the network.
- Average dissipated energy in an important metric. This is the ratio of total energy used per node to the number of events detected.
- A useful evaluation metric list includes “Resource expended per packet delivered” metric. Here “resource expended” refers to the numbers of connected pairs that are broken down because of nodes being depleted on its power. In other words, it is defined as the ratio between numbers of broken pairs to the total packets delivered.
- The metric “packets before partition” is measured by the number of data packets sent and successfully delivered before network partition (partition due to node energy depletion).

### 3.2. Evaluation Measures Related to Lifetime of Network

The evaluation metric “lifetime of the network” has a strong dependence on the nodes' battery capacity. As such, the network lifetime has been a critical concern in WSN research. While numerous energy-efficient protocols have been proposed to prolong the network lifetime, various definitions of network lifetime have also been used for the different scenarios and protocols. The lifetime of a sensor network is most commonly defined as the time to the first sensor node failure. However, this definition is seemingly over-pessimistic in many envisaged deployment scenarios, such as habitat monitoring, forest fire detection *etc.* [16]. While other definitions exist, there has not been any consensus on which quantitative lifetime definition is most useful. The various “network lifetime” definitions proposed and used in the literature include:

- Time till the first sensor node failure [17–20].
- Time till certain percentage of sensor nodes failure, or surviving nodes in the network (falls below a given application-dependent threshold) [21,22].
- Time till the network becomes disjoint; network partitions emerge [23].
- Time till size of the largest connected component drop below a threshold [24].
- Time till the packet delivery rate falls below a certain threshold [25].
- Time till all the sensor nodes dies [26].
- Time till number of errors exceeds a threshold [27].
- Time till the number of packets that can be (successfully/correctly) delivered by the network falls below a threshold [28].

### 3.3. Evaluation Measures Related to Scalability

Scalability of a network means that the protocols running in the network perform well as the network grows larger or as the workload increases [29]. Scalability is an important factor in designing routing protocols for WSN. A good routing protocol has to be scalable and adaptive to the changes in the network topology. Routing packets within a large scale WSN without storage overhead and routing table updates is a challenging problem. To constrain this communication overhead, routing in sensor network demands efficient protocols for routing packets.

For large-scale WSNs, scalability is an important metric that measures how a protocol performs at varying node density, overall network size, or number of data sources and sinks. Many researchers have tried to establish mathematical models for scalability, and using these mathematical models, they put some numerical values against this qualitative metric to compare different protocols/architectures.

### 3.4. Evaluation Measures Related to Overhead and Efficiency

In conjunction with the direct measures of energy, the other metrics related in evaluating the performance are overheads and efficiency of the WSN. The following is a list of possible metrics.

- *Routing Protocol Message Cost*. This is one of the most common metrics used for evaluating the efficiency of the protocols. It measures the number of packets generated by a protocol/algorithm for each successful communication.
- *Message Loss*. It measures the percentage of messages not received by any node in the network.
- *Control Overhead*. It measures the ratio between control and data messages being transmitted in the network. Some authors also use the overall packets sent or packets received and others compare the application packet delivery rate with the routing packet rate.
- *Event Delivery Ratio*. This criterion is the ratio of the number of distinct event messages received by the sink to the number originally sent by the source. Some authors measure a related “loss to collision” ratio.
- *Transmissions to Query Ratio*. This is the ratio between the total number of packets to the number of queries injected into the sensor network.
- *Average Path or Route Length*. It measures the number of hops from source node to destination node. Although it is related to energy usage, each path or route can give very different results due to the nonlinear relationship between transmission power and range.

### 3.5. Temporal Evaluation Criteria

The primary temporal evaluation criteria used in the existing literature are the latency and the reaction time.

- *Latency*. This is measured by the average delay between transmitting an event message and receiving it at the sink. There have also been other measures used by many researchers to calculate the latency. One way is to calculate the total time elapsed to perform an action by the sensor nodes, for example, disseminate information to a set of nodes, or to complete a number of data collection rounds. Another way to calculate the latency is to calculate the time duration for identifying an event or to reach a consensus for a measured value.
- *Reaction Time*. The definition of this term varies among researchers, but essentially captures the average time it takes for the sink to receive data or particular messages after some change occurs in the network.

### 3.6. Other Performance Evaluation Measures

The other various measures used in the existing literature of WSNs, which are concerned about deployment and design related issues, are mentioned below.

- *Storage Requirement*. This is measured by the amount of memory required by an algorithm at each node.
- *Ease of Deployment*. This metric is mentioned by some researchers in their technical papers but no specification of this metric has been found.

## 4. Descriptions of Different Topologies of WSNs

This section identifies and studies various types of topologies of WSNs. First of all, different topologies of WSNs are identified. In doing so, different application protocols proposed by various researchers, such as protocols for data gathering/collection, target tracking, routing, data aggregation, data dissemination, *etc.*, are studied. These protocols use various types of logical topologies. From each of the protocols, the topology is identified and listed in Table 1. The identified topologies are (i) flat topology, (ii) cluster-based topology, (iii) chain-based topology and (iv) tree-based topology.

The following subsections describe each of the topologies with their advantages and disadvantages in detail.

### 4.1. Flat/Unstructured Topology

**Figure 2.**
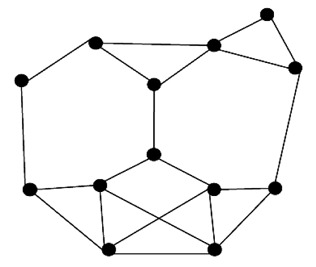
Flat topology architecture.

This is actually the case of no topology or the absence of any defined topology. In flat topology, each sensor plays equal role in network formation. Different protocols have been proposed based on flat/unstructured topology. For example, this flat topology has been used in data aggregation protocols [53], data gathering protocols [30], node scheduling protocols [66], and routing protocols [67]. Figure 2 shows the flat topology architecture where the nodes are the sensors and the edges are available communication links between two sensors.

All the protocols, while using flat topology, attempt to find good-quality routes from source nodes to sink nodes by some form of flooding. Flooding is a technique in which a given node broadcasts data and control packets that it has received to the rest of the nodes in the network. This process repeats until the destination node is reached. Note that, this technique does not take into account the energy constraints imposed by the WSNs. As a result, when used for data routing in WSNs, it leads to the following two problems, namely, implosion and overlap [44]. Given that flooding is a blind technique, duplicate packets may keep circulate in the network, and hence sensors will receive those duplicate packets, causing an implosion problem. Also when two sensors sense the same region and broadcast their sensed data at the same time, their neighbours will receive duplicated packets.

In a flat network, data aggregation is accomplished by data-centric routing where the base station usually transmits a query message to the sensor nodes via flooding, and the sensor nodes that have data matching in the query will send response messages back to the base station. The sensor nodes communicate with the base station via multi-hop routes by using peer nodes as relays. The choice of particular communication protocol depends on the specific application.

Since flooding is a very costly operation in resource starved networks, smart routing algorithms restrict the flooding to localized regions. Some algorithms use probabilistic techniques based on certain heuristics to establish routing paths. Some examples of routing protocol based on flat topology are Sensor Protocols for Information via negotiation (SPIN) [44,68], Directed-Diffusion [69], Rumor-Routing [70], *etc*.

SPIN's meta-data negotiation solves the classic problems of flooding to some extent. SPIN is a three-stage protocol as sensor nodes use three types of messages, such as ADV, REQ, and DATA to communicate sensors with each other. ADV is used to advertise new data, REQ is used to request data, and DATA is the actual message itself. The protocol starts when a SPIN node obtains new data it is willing to share. It does so by broadcasting an ADV message containing metadata. If a neighnour is interested in the data, it sends a REQ message for the DATA and the DATA is sent to this neighnour node. The neighnour sensor node then repeats this process with its neighnours. As a result, the entire sensor area will receive a copy of the data. One of the advantages of SPIN is that topological changes are localized since each node need know only its single-hop neighnours. SPIN provides more energy savings than flooding, and metadata negotiation almost halves the redundant data. However, SPIN's data advertisement mechanism cannot guarantee delivery of data. To see this, consider the application of intrusion detection where data should be reliably reported over periodic intervals, and assume that nodes interested in the data are located far away from the source node, and the nodes between source and destination nodes are not interested in that data; such data will not be delivered to the destination at all.

Directed diffusion differs from SPIN in two aspects. First, directed diffusion issues data queries on demand as the BS sends queries to the sensor nodes by flooding some tasks. In SPIN, however, sensors advertise the availability of data, allowing interested nodes to query that data. Second, all communication in directed diffusion is neighnour to neighnour with each node having the capability to perform data aggregation and caching. Unlike SPIN, there is no need to maintain global network topology in directed diffusion. However, directed diffusion may not be applicable to applications (e.g., environmental monitoring) that require continuous data delivery to the BS. This is because the query-driven on-demand data model may not help in this regard. Moreover, matching data to queries might require some extra overhead at the sensor nodes.

Rumor routing performs well only when the number of events is small. For a large number of events, the cost of maintaining agents and event tables in each node becomes infeasible if there is not enough interest in these events from the BS. Moreover, the overhead associated with rumor routing is controlled by different parameters used in the algorithm such as time to live (TTL) pertaining to queries and agents. Since the nodes become aware of events through the event agents, the heuristic for defining the route of an event agent highly affects the performance of next-hop selection in rumor routing [56].

Overall, the advantages of flat-based protocols include (a) good quality routes from source to sink, and (b) no topology maintenance overhead.

On the other hand, there are few disadvantages:

- The main way of communication is flooding. However, flooding is an expensive operation that is normally avoided by sensor network routing protocols.
- A large number of redundant messages are created and passed. This redundancy consumes processing cycles as well as bandwidth of the network. Due to the redundancy latency increases because of the high contention of wireless communication medium.
- Non-uniform energy distribution occurs in flat/unstructured topology. This is the reason why the lifetime of a sensor network decreases.
- Sensors are not aware of new members or died members.
- Highly unreliable.
- High delay.

### 4.2. Cluster-Based Topology

Cluster-Based topologies have widely been used in WSNs for various types of protocols, such as data gathering [31], target tracking [38], one-to-many, many-to-one, one-to-any, or one-to-all communications, routing [26,35,45,47], *etc*. Clustering is particularly useful for applications that require scalability to hundreds or thousands of nodes. Scalability in this context implies the need for load balancing, efficient resource utilization, and data aggregation. Many routing protocols also use clustering to create a hierarchical structure and minimize the path cost when communicating with the base station.

#### 4.2.1. Elements in a Cluster

**Figure 3.**
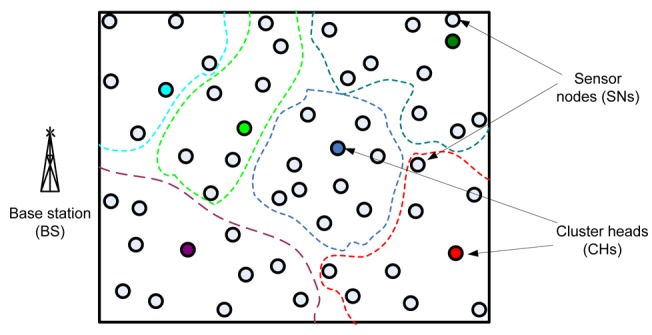
Cluster-based topology architecture. Dotted line boundaries refer to a cluster.

In general, when working with clusters it is possible to identify three main different elements in the WSN: sensor nodes (SNs), base station (BS) and cluster heads (CH) (see Figure 3). The SNs are the set of sensors present in the network, arranged to sense the environment and collect the data. The main task of a SN in a sensor field is to detect events, perform quick local data processing, and then transmit the data. The BS is the data processing point for the data received from the sensor nodes, where the data is accessed by the end-user. It is generally considered fixed and at a large distance from the sensor nodes. The CH acts as a gateway between the SNs and the BS. The function of the cluster head is to perform common functions for all the nodes in the cluster, like aggregating the data before sending it to the BS. In some way, the CH is the sink for the cluster nodes, and the BS is the sink for the cluster heads. This structure formed between the sensor nodes, the sink and the base station can be replicated as many times as it is needed, creating the different layers of the hierarchical WSN. The greatest constraint it has is the power consumption, which usually is caused when the sensor is observing it surroundings, and communicating (sending and receiving) data.

#### 4.2.2. Cluster Types

There exist many different ways to classify the clusters. Two of the most common classifications are homogeneous or heterogeneous clusters and static or dynamic clusters. The former classification is based on the characteristics and functionality of the sensors in the cluster, whereas the later is based on the method used to form the cluster.

In homogeneous networks, all nodes have the same characteristics, hardware and processing capabilities. The cluster head role is periodically rotated among the nodes to balance the load, ensure that sensors consume energy more uniformly, and try to avoid the black hole problem.

In heterogeneous sensor networks, there are generally two types of sensors:

- Sensors with higher processing capabilities and complex hardware, used generally to create some sort of backbone inside the WSN. They are designated as the cluster head nodes, and therefore have to serve as data collectors and processing centres for data gathered by other sensor nodes.
- Participating sensors, with lower capabilities than the previous ones, used to actually sense the desired attributes in the field.

Static clusters are usually created when the network is formed of heterogeneous nodes and the network designers want to create the clusters around the more powerful nodes. In this case, the clusters are formed at the time of network deployment. The attributes of each cluster, such as the size of a cluster, the CH, the number of participating sensors and the area it covers, are static. Static clusters are easy to deploy, but their use is only appropriate for limited scenarios where the sensor field is predetermined, the targets to monitor are not in motion and it is easy to perform maintenance tasks (*i.e.*, sensors replacements) in the network. On the other hand, Dynamic cluster architectures make a better use of the sensors. Sensors do not statically belong to a cluster and may support different clusters at different times.

#### 4.2.3. The Clustering Process

During the establishment of the cluster, it is necessary to take into account aspects like: cluster size and form, how to select the cluster head, how to control inter-cluster and intra-cluster collisions, and energy saving issues. The design of the clustering process is one of the more important issues for the correct functioning of the WSN, due to the probed efficiency of using a hierarchical scheme for communications between the network elements.

In all the cluster-based protocols, three main phases can be identified during the clustering establishment process: (a) cluster head election phase, (b) cluster formation or set up phase, and (c) data transmission phase (steady-state phase). Different approaches exist to implement each one of these stages. For example, it is possible to use a fixed distribution of the SN and the CH, or to use a dynamic algorithm for the location of the sensors and the CH election. Clusters may be formed in any one of the following ways:

- *Probabilistic Method*: LEACH protocol uses this method where each sensor randomly picks a real number from 0 to 1. If the number is greater than a threshold value, the sensor declares it as a cluster leader and broadcasts invitation messages to all other sensors. A sensor, not picking a real number greater than threshold joins any one of the leaders. Thus clusters are formed.
- *By Election Phase*: In this method all the sensors broadcast their information to all other sensors and form a knowledge base. Based on the local knowledge they form cluster and then select a leader.
- *Assigned by the Base Station*: In this method, clusters are formed by the base station. After deployment of the sensors, all nodes communicate with base station and based on the information in the base station, it tries to form optimal nodes. Although this method can form optimal clusters, this method is rarely used because of the cost incurred for the communications of all sensors to the base station. An example protocol of this is the BCDCP [10] (Base station Controlled Dynamic Clustering Protocol).

#### 4.2.4. Advantage and Disadvantage of Cluster-Based Topologies

Cluster-based routing protocols greatly increase the scalability of a sensor network. The overall energy consumption of the nodes compared with the flat topology protocols is reduced, leading to prolonged network lifetime. The organization of the network into clusters lends itself to efficient data aggregation, which in turn results in better utilization of the channel bandwidth. Cluster-based routing holds good promise for many-to-one and one-to-many communication paradigms that are prevalent in sensor networks. However, non-uniform clustering is the main problem for this topology. Consider the LEACH protocol, there is a fair chance that most of the cluster heads are situated in a same side of the network where as few cluster heads on the other side or even worsen no cluster head in a specific area. Thus, non-uniform clustering happens. Due to non-uniform clustering, the following problems occur:

- Energy dissipation rate is highly different from one sensor to another sensor, even if they are in the same cluster. Thus energy distribution is not even.
- Total energy dissipation increases due to the long way communication between a cluster member and cluster head.
- Because of very long-way communications, some sensors consume energy rapidly and die. As a result, network lifetime decreases.
- Network connectedness may not be guaranteed.

### 4.3. Chain-Based Topology

In this topology, the protocols construct transmission chain(s) connecting the deployed sensor nodes to save energy dissipation of data transmission. A leader is selected in a chain that acts as the sink. All sensor nodes communicate with each other along the chain. A node sends data to the next node, which is called successor node of the former node, towards the leader node. A successor node, receiving data from the predecessor node, forwards the data to its successor node towards the leader. In this fashion, all sensor nodes send their sensed data to the leader node(s). This way of communication facilitates the data aggregation.

PEGASIS [71] is an example protocol based on chain topology. In PEGASIS, every node in chain senses the data, receives data from its predecessor, fuses with received the predecessor's data and transmits to next node in chain. Data aggregation performs in-network fusion of data packets, coming from different sensors en-route to the base station, in an attempt to minimize the number and the size of data transmissions and thus save sensor energy.

The basic idea of the PEGASIS protocol is that in order to extend network lifetime, nodes only need to communicate with their closest neighnours, and they take turns in communicating with the BS. When the round of all nodes communicating with the BS ends, a new round starts, and so on. This reduces the power required to transmit data per round as the power draining is spread uniformly over all nodes. Hence, PEGASIS has two main objectives. First, increase the lifetime of each node by using collaborative techniques. Second, allow only local coordination between nodes that are close together so that the bandwidth consumed in communication is reduced. The chain construction is performed in a greedy fashion. Simulation results showed that PEGASIS is able to increase the lifetime of the network to twice that under the LEACH protocol. Such performance gain is achieved through the elimination of the overhead caused by dynamic cluster formation in LEACH, and decreasing the number of transmissions and reception by using data aggregation.

Although the clustering overhead is avoided, the protocol PEGASIS still requires dynamic topology adjustment since a sensor node needs to know about the energy status of its neighnours in order to know where to route its data. Such topology adjustment can introduce significant overhead, especially for highly utilized networks. On the other hand, PEGASIS introduces excessive delay for distant nodes on the chain. In addition, the single leader can become a bottleneck. Finally, although in most scenarios sensors will be fixed or immobile as assumed in PEGASIS, some sensors may be allowed to move and hence affect the protocol functionality.

**Figure 4.**
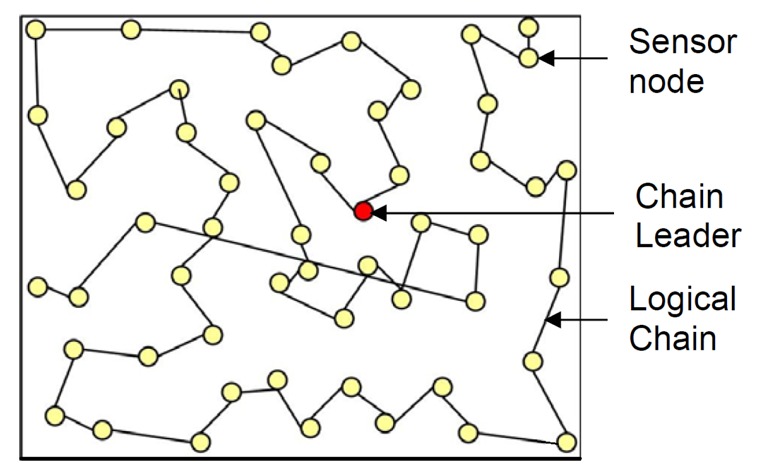
Chain-oriented topology architecture (used in PEGASIS).

Figure 4 shows the chain-based topology used in PEGASIS. The circles represent sensor nodes whereas a bidirectional line between two nodes represents successor-predecessor relationship.

Besides PEGASIS, there are also other protocols such as COSEN and CHIRON that use chain-based topologies. COSEN is probably the first chain-oriented topology that used multiple chains instead of a single chain. Here we summarize the advantages and disadvantages of chain-based topology

Here advantages and disadvantages of chain-oriented topology are summarized.

#### Advantage

- Chain-oriented topology saves more energy than cluster-based topologies do. For example, PEGASIS saves 50% more energy compared with LEACH citepchap 4-16.
- Energy distribution in a chain-oriented topology is even
- Because of better energy conservation, chain-oriented topologies offer longer lifetime for WSNs.

#### Disadvantages

- Too much delay for data collection.
- Topology management overhead is high.

### 4.4. Tree-Based Topology

**Figure 5.**
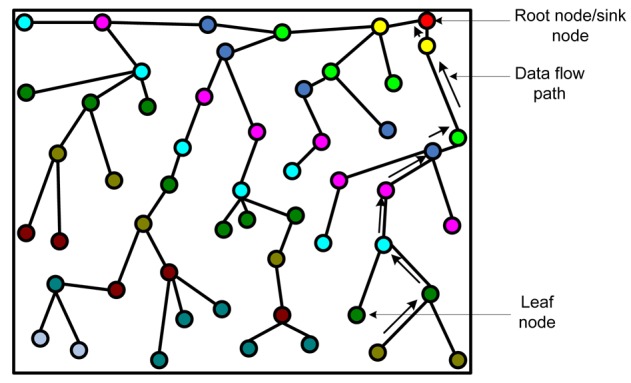
Tree-Based topology architecture.

In this topology, all the deployed sensors construct a logical tree. Data are passed from a leaf node to its parent nodes. In turn, a receiver node receiving data from the child node sends data to receiver's parent node after aggregating data with its own data. In this fashion, data flow from leaf nodes to the root node, which generally acts as the sink. The idea behind constructing logical tree is that it avoids flooding and data can be sent using unicast instead of broadcast. This way the topology can save energy. Figure 5 shows a typical formation of logical tree. The arrows show the data flow from a leaf node to the root node/sink.

Tree topology is used to design various protocols for WSNs, such data collection scheme (TBDCS [34]), routing protocols ([72,73]), data dissemination protocols ([56,57]) *etc*.

One of the advantages of this topology is that it consumes less power than flat topology, as flooding is not necessary for data communication. Furthermore, it can save a bit more energy than some protocols based on cluster topology. In [74], Zhang *et al.* prove that for data acquisition, tree-based topology saves more energy than cluster-based topology.

The disadvantages of tree-based topology are:

- Formation of tree is time consuming and costly.
- It is not resilient to node failures. If a parent node fails, then its entire sub-tree is cut off from the base station during the current epoch.
- Power consumption is uneven across network nodes. The nodes closer to the base station consume a lot of power in forwarding packets from all the nodes in their sub-tree, whereas the leaf nodes in the spanning tree do not have to perform any forwarding at all and consume the least power.
- Long delay for sending data from leaf to root node.
- Tree maintenance overhead is high.

## 5. Comparison of Different Topologies

This section compares the different topologies introduced above, namely flat, cluster-based, chain-based and tree-based topologies. They will be compared using the performance metrics that is described in Section 3.

### 5.1. Topology Comparison Based on Energy Efficiency

Energy efficiency is the most important constraint and performance metric for WSNs due to the limited energy resources of the sensor nodes and their operations in unattended and inaccessible environments where replacement of energy resources might be impossible. Therefore, while traditional networks aim at achieving high quality-of-service provisions, WSNs focus primarily on energy awareness in every aspect of hardware and software design and operations to prolong the useful lifetime of each sensor node and, more importantly, of the entire WSN [75].

**Figure 6.**
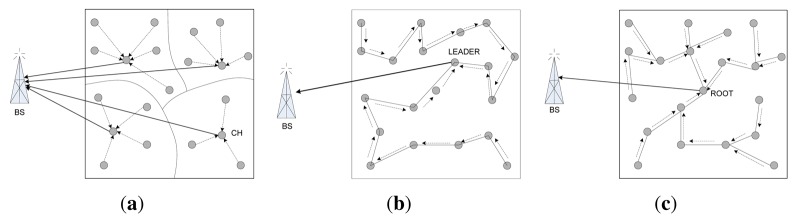
Communication patterns for different topologies of WSNs. (**a**) cluster topology, (**b**) chain topology, and (**c**) tree topology.

Communication is the most energy intensive activity performed by the sensor nodes, and hence the WSN topology and communication protocols can play a significant role in the energy efficiency and lifetime of the WSN. Figure 6 depicts the communication patterns of basic cluster, chain and tree topologies. The energy required for communication scales with distance between two nodes (*d*) from *d*^2^ to *d*^4^. Since the radio signal attenuation scales with distance in a greater-than-linear fashion, the multi-hop communication in chain topology consumes less power than the single-hop long distance radio communication in cluster topology [76]. In chain-based topologies, chains are usually formed considering the minimum distance from a node in the chain to its successor. On the other hand, while configuring clusters, distance has never been used as a selection criterion. Simulation results show that summation of d2 values is the minimum for a chain-oriented topology compared with cluster-based or tree-based topologies. As the total energy consumption is directly proportional to the *d*^2^ / *d*^4^, total energy consumption for chain-based topology is always lower than other topologies. For example, PEGASIS spends only 70% of total energy spent by LEACH for 300 rounds of data collection [71].

Energy consumption of the sensor nodes of a WSN should be evenly distributed. If some node spends too much energy to perform a task, by repetition of that task, those nodes would lose their energy rapidly and die soon. It is apparent from Figure 6 that, in cluster and tree topologies, CHs (cluster topology) and parents nodes (tree topology) handle more traffic than the leader node(s) of chain topology. As a result, those nodes in cluster and tree topologies deplete their energy faster than other nodes, and thereby disconnecting the base station from the whole WSN, which might still have adequate resources and infrastructure. This is the well-known self-induced black hole effect. Simulations shows that nodes closest to the base station are the ones to die out first for flat mesh routing, whereas nodes farthest from the base station are the ones to die out first for direct transmission [77].

In-network processing, which is primarily based on the motivation that typically computation is more energy-efficient than communication, is one of the key mechanisms with the potential to considerably improve the energy efficiency of WSNs. Simulations have shown that it typically requires around 100 to 1000 times more energy to transmit a bit than to execute an instruction [78]. Possibilities for in-network processing in WSN include aggregation and compression, which exploit spatial and temporal correlation in the sensed data, for performing local compression to reduce global communication to base station by reducing the overhead of packet headers, by compressing the payload, and by reducing the probability of packet collisions. For example, the predominant communication in WSN is converge-cast, *i.e.*, collection of sensed data from multiple sensors at the base station: a kind of reverse multicast. In addition, coverage-cast aligns closely with the need and capacity of WSN to perform in-network processing.

In a flat topology, routing paths are not fixed a priori, and hence the opportunity for the in-network processing is very much limited. In a cluster-based topology, where the cluster members reach the cluster head in a single hop, only the cluster heads can be used for data aggregation/pre-processing. For a tree-based topology, sensor nodes get more opportunity to aggregate/pre-process data. For example, a parent node can process the data received from its child node(s). Finally, chain-based architecture is inherently amenable to in-network processing. In this topology, each single node can be used for in-network processing of its predecessor's data. It decreases communication traffic and communication frequency via data aggregation progressively at each leader in the chain by processing and filtering the possibly redundant data received from other chain members. Unlike the cluster topology, the leader of a chain is not responsible for all data aggregation. Every member of a chain participates in the process of data aggregation. This actually balances the load among the nodes of a chain and thus energy consumption is evenly distributed. This is one of the important reasons why chain-based topology offers longer lifetime of WSNs.

In summary, chain-based topology performs best regarding energy efficiency. On the other hand, flat topology is the least energy efficient topology. Cluster-based topology is ahead of tree-based topology with respect to energy efficiency. However, the energy efficiency of cluster-based topology primarily depends on the cluster formation algorithm.

### 5.2. Topology Comparison Based on Reliability

Reliability analysis is an important task for the successful operation of WSNs. IEEE P1451.5 web survey [56] identified data reliability as one of the most important parameters in the design of WSNs. Reliability is generally defined as the probability that the system will perform its intended function under stated conditions for a specified period of time [79].

The WSN reliability can be studied for three different scopes of data delivery [80], collectively known as the infrastructure communication: (a) users send their interest to a single sensor node, (b) users send their interest to a subset of nodes in a sub-area and the message needs to be delivered to all sensors in the particular group, and (c) users send their interest to the entire sensor network and the message needs to be delivered to all sensors in the network. There exists another scope of message delivery, known as application communication, in which it is sufficient that the message from sink is reliably delivered to only a group of sensor nodes that together cover the entire sensor field or the intended area of observation [78]. This is different from the delivery to all sensors in the infrastructure communication, due to the typical redundant deployment of sensors.

Given a single node failure, the flat topology reduces the chance of the entire network failure, because the failure of any node results only in the localized failure, leaving the rest of the system unaffected. However, when a node becomes obstructed, there is no alternate path from the associated node to the base station. A flat topology is highly fault-tolerant as it offers multiple redundant paths throughout the network. If a routing node fails or the link between nodes becomes unavailable, the network can automatically reconfigure itself around the failed component. In a WSN, the degree of redundancy, and in general the reliability of the network, is essentially a function of node density. In addition, a WSN with flat topology can be deliberately over-provisioned for reliability simply by adding extra nodes. The addition of redundant nodes also improves the reachability of WSN by providing multi-hop routes to inaccessible or hidden nodes. Besides, if certain environmental or architectural conditions results in poor reliability, it is difficult or impossible to adapt a point-to-point network like the network with a cluster topology to increase reliability. In contrast, the WSN reliability can be improved by redeploying redundant nodes in the affected area.

The tree topology has the lowest reliability due to the use of only a single direct link between nodes at successive levels in the hierarchy. Chain-oriented topology offers better reliability than tree-based topologies as in the same hierarchy level it uses multi-hop communication from source to sink. Clustered hierarchical topology is a compromise between the two extremes. It is better than tree/chain-oriented topology, as it maintains multi-hop paths, while it has lower reliability than flat topology because each communication between nodes at different clusters must route through affiliated cluster heads. This is to note that, in WSNs, the residual energy of a node affects the reliability in an indirect way. For example, if the energy of the cluster head goes down, then the reliability decreases in an exponential manner. Moreover, energy simultaneously affects the number of normal and critical faults. As the energy decreases, the number of faults increases [81]. As energy efficiency of chain-based topology is higher than that of any other topology, it is possible that energy efficiency of chain-oriented topology compensates the relatively weak reliabilities.

### 5.3. Topology Comparison Based on Scalability and Self-Organization

WSN should be scalable to varying sensor density, and should maintain performance that is independent of the number of nodes or gracefully degrade the performance depending on the number of surviving nodes. In addition, WSN will presumably be required to self-configure into connected networks, and will require different or at least adaptive protocols. For example, by allowing the algorithms and protocols to trade off accuracy and latency with energy dissipation, WSN can be scalable and flexible to the application requirements that might change over the WSN lifetime.

Self-organization helps in maximizing the network lifetime. Nevertheless, self-organization should be kept in perspective with energy cost and speed. Sometimes letting a WSN to kill its nodes may be more energy efficient than trying to revive it [82]. Self-configuration time involves fault identification, fault localization and fault recovery phases. The self-configuration time for tree topology can be quite high, while that for cluster and chain-oriented topology is less compared with the time required for tree-based topology. In the chain-based and clustered hierarchical approaches, the chain leader and the CH respectively can initiate the localized reconfiguration of the chains and clusters.

In flat topology, high number of sensor nodes increases load on the base station, which results in increased power consumption and complexity. In addition, as node density increases, the increase in collisions greatly degrades performance. Further, not all nodes have enough transmission range or the line-of-sight communication with the base station. It is difficult to scale flat WSN to more than a few nodes.

For tree-based topologies, self-configuration and scalability are limited up to a certain number of depths of the tree. After that, WSN designers should carefully plan the transmission and duty cycle scheduling of the sensor nodes to avoid aforementioned negative effects of dense deployment. Therefore, in practice, tree-based topology works well for medium sized networks, but has scalability limitations that degrade performance for larger or densely deployed WSN.

The scalability and self-organization issues of chain-based topology primarily depend on the number of chains in the network. Chain-oriented topology with a single chain (PEGASIS) has the same limitations that the tree-based topologies have. Multiple chain-based topology and clustered hierarchical topologies improve the scalability of the flat networks by assigning leaders and/or cluster heads to manage the local neighbourhood of sensor nodes. For example, LEACH and COSEN use localized coordination to enable scalability and robustness for dynamic networks [56,57]. In addition, the adaptive self-organizing capabilities of multi-chain and clustered hierarchical WSNs allow the periodic reformation of hierarchical chain/clusters of sensor nodes, in the event of environmental or topological changes when sensor nodes fail or new sensor nodes are added to improve connectivity and coverage.

Besides, for scalability, the addressing structures of WSN are likely to be quite different; for example, geographic, data-centric, or address-free structures. Distributed and/or probabilistic assignments of addresses are only unique in a two-hop neighbourhood. For example, address-free architecture [81], which leverages the spatial and temporal locality of WSN to assign probabilistically unique identifiers for each new transaction, must only scale with the transaction density of WSN, while a statistically assigned global address space must scale with the total number of nodes in the WSN. Hierarchical tree and hierarchical clustered architectures are inherently amenable to a scalable addressing structure where the nodes are addressed based on their position in the hierarchy. For example, a node z that is a member of level-1 cluster y and level-2 cluster x could have an address “x.y.z” [83]. This scheme also allows simple routing protocols with small footprint that are scalable and occupy small memory space.

### 5.4. Topology Comparison Based on Data Latency

The WSN traffic, which is characterized by the interaction with the environment or generated in response to certain events, is likely to be very different from human-driven forms of networks. A typical consequence is that WSNs are expected to exhibit very low data rates over a large time scale, but can have very burst traffic upon the occurrence of the certain events.

A single-hop-to-sink structure would have the least data latency because there is no delay due to buffering at routers along the path. However, it is not scalable and there may be more loss due to collisions as the network density increases. Flat topology networks have higher data latency than the single-hop-to-sink structure but lower data loss because keeping the transmission power lower reduces the packet collision rates. Depending on the number of nodes and the distance between them, flat topology network may endure increased latency as message moves along multi-hop route to the base station. In addition, a flat topology network can cause the nodes closer to the base station to overloads with the increased node density. Such overload might cause latency in communication, and the worst case creates a black hole of overloaded (or dead) nodes around the base station.

In hierarchical tree topology, as the data moves from the lower level to a higher level, it moves a greater distance, thus reducing the travel time and data latency. However, as the distance cluster levels increases, the energy dissipation, which is proportional to square of distance, increases. In [71], Lindsey *et al.* propose a metric by “energy × delay” and presents a chain-based scheme called PEGASIS that attempt to balance the energy and delay cost for data gathering from WSN. Clustering is a design approach to minimize energy consumption and to minimize data latency. In clustered hierarchical topology, only CH (along the hierarchy) performs aggregation, while in chain topology intermediate nodes perform aggregation. As a result, clustered hierarchical architecture has lower latency than chain topology. Nevertheless, individual packet latency may not be important criteria due to the inherent redundancy (caused by spatial and temporal correlation in the sensed data) in the transmitted packets.

### 5.5. Topology Comparison Based on Overhead and Efficiency

Flat topology produces the maximum number of packets for routing. In a flat topology, because of flooding, a node can receive multiple copies of same data from different nodes. On the other hand, in cluster-based topology, the cluster heads receive data from all members of the cluster. In tree topology, a parent node receives data from its children node(s). However, in a chain-based topology, a node in a chain receives data from only one node. Thus, in terms of communication overhead, chain-based topology is the best, tree-based topology is the second best, cluster-based topology is good, and flat topology is bad.

In terms of control overhead, which measures the ratio between control and data messages in the network, flat topology is the best because it does not need to maintain any structure. Thus, this topology does not need to disseminate topology control messages. Tree-based topology, on the other hand, has the largest number of control message overhead to maintain the tree structure. Cluster-based and chain-based topologies also have control message overheads, but much less than that of the tree-based topology.

### 5.6. Topology Comparisons at a Glance

Table 2 summarizes the comparative analysis of the four topologies based on the assumptions described in Section 2. The topologies were compared with ten performance metrics, namely total energy consumption, energy distribution, load distribution, redundant communication, data reliability, scalability, latency, network connectedness, lifetime, and topology management overhead. We mark each topology out of four for each evaluation metric according to their performance with regard to the corresponding metric. Finally, the points for each evaluation metrics of each topology are added. The totals, which indicate the overall performance, for each topology are shown at the bottom most row of the table.

In our point system, 4 means excellent, 3 means good, 2 means fair and 1 means poor. Depending on the design structure of a topology, some fields of the table have a range of numbers presented as x to y. For example, with regard to latency, single chain-based topology shows fair results, whereas multi-chain topology shows excellent results. Thus for latency, the chain-based topology received 2 to 4. These points used here are entirely relative. For example, with respect to energy consumption, cluster-based topology is better than flat topology but not as efficient as chain-based topology. Note that this point system is used for the purpose of easy understanding and does not provide a standardized measure for comparison.

The table shows that the chain-oriented topology scores the highest among four topologies, whereas the flat topology scores the lowest. The cluster-based topology performs better than the tree-based topology but not as efficiently as the chain-based topology. Nevertheless, there are some areas in the chain-based topologies to which special attentions should be paid by the designer to make the topology more efficient.

## 6. Conclusions

In this paper, different topologies, which are used for designing different protocols by the researchers, are identified. The topologies, namely flat, cluster-based, chain-based, and tree-based topologies, are discussed in detail. This paper also discusses different performance metrics of WSN topologies. Defining a system model, all topologies are compared against each other using these performance evaluation metrics. The summary of the comparison of different topologies using 10 performance metrics is provided in Table 2. It is to be noted that, the numbers used in this table is not absolute; rather they are relative to each other.

From the discussion of this paper, it can be argued that chain-oriented topology is the most promising topology among all topologies described in this paper. Moreover, there are some provisions to make the chain-oriented topology perform even better. As a result, we would like to continue the research in constructing efficient chain-based topologies.

## Figures and Tables

**Table 1.** Different protocols and their corresponding topology.

Protocol for	References	Topology used in the protocol
Data gathering	[30]	Flat
	[31]	Cluster-based
	[32–34]	Tree-based
	[35,36]	Chain-based
Target Tracking	[37]	Flat
	[38]	Cluster-based
	[39–41]	Tree-based
	[42]	Chain-based
Routing	[43,44]	Flat
	[26,45–47]	Cluster-based
	[38,48–50]	Tree-based
	[51,52]	Chain-based
Data aggregation	[53]	Flat
	[54,55]	Cluster-based
	[56,57]	Tree-based
	[58]	Chain-based
Data dissemination	[59]	Flat
	[60]	Cluster-based
	[61]	Tree-based
	[62]	Chain-based
Synchronization	[63]	Flat
	[64]	Cluster-based
	[34,65]	Tree-based

**Table 2.** Comparison of different topologies.

Evaluation metric	Flat	Cluster-based	Tree-based	Chain-based
Total energy consumption	1	3	2	4
Energy Distribution	1	3	2	4
Load distribution	3	3	3	4
Redundant Communication	1	4	4	4
Data reliability	4	3	3	2 to 3
Scalability	2	4	3	2 to 4
Latency	4	3	3	2 to 4
Network Connectedness	1	3	3	3
Lifetime	2	3	3	4
Topology management overhead	4	3	2	4

Overall Scores	23	32	28	32 to 37
